# Depot-specific inflammation with decreased expression of ATM2 in white adipose tissues induced by high-margarine/lard intake

**DOI:** 10.1371/journal.pone.0188007

**Published:** 2017-11-15

**Authors:** Nannan Wang, Jie Guo, Fuding Liu, Mingxia Wang, Chuntao Li, Lihong Jia, Lingling Zhai, Wei Wei, Yinglong Bai

**Affiliations:** 1 Department of Child and Adolescent Health, School of Public Health, China Medical University, Shenyang, Liaoning, China; 2 Department of Medical Statistics and Epidemiology, School of Public Health, Sun Yat-sen University, Guangzhou, Guangdong, China; 3 Department of Public Health, Huangshi Central Hospital, Edong Healthcare Group, Huangshi, Hubei, China; 4 Information Center, the First Hospital of China Medical University, Shenyang, Liaoning, China; Hospital Infantil Universitario Nino Jesus, SPAIN

## Abstract

A high-fat diet has been recognized as an important risk factor of obesity, with variable impacts of different fatty acid compositions on the physiological process. To understand the effects of a high-margarine/lard diet, which is a major source of trans fatty acids (TFAs)/ saturated fatty acids (SFAs), elaidic acid as a biomarker of margarine intake was used to screen affected adipokines on mature human adipocytes in vitro. Weaned male Wistar rats were fed a high-fat diet enriched with margarine/lard to generate obesity-prone (OP) and obesity-resistant (OR) models, which were then used to explore the inflammatory responses of depot-specific white adipose tissue. Adiposity, glucose and lipid metabolism parameters and macrophage cell markers were also compared in vivo. In the subcutaneous depot, a high-margarine diet induced elevated IL-6, MCP-1 and XCL1 expression levels in both M-OP and M-OR groups. High-lard diet-fed rats displayed higher protein expression levels of MCP-1 and XCL1 compared with the control group. In the epididymal depot, significantly elevated IL-6 production was observed in M-OP rats, and high-lard diet-fed rats displayed elevated IL-6 and decreased XCL1 expression. In the retroperitoneal depot, a high-margarine diet caused higher IL-6 and MCP-1 expression levels, a high-lard diet caused elevated IL-6 expression in L-OP/L-OR rats, and elevated XCL1 expression was observed only in L-OP rats. In general, CD206 mRNA levels were notably down-regulated by high-fat diet feeding in the above-mentioned depots. CD11c mRNA levels were slightly upregulated in the subcutaneous depot of OP rats fed a high-margarine/lard diet. In the epidydimal depot, higher expression levels of F4/80 and CD206 mRNA were observed only in high-margarine diet-fed OP rats. These results suggest that depot-specific inflammation with decreased expression of adipose tissue anti-inflammatory M2-type (ATM2) macrophages could be induced by high-margarine/lard intake.

## Introduction

Overweight and obesity are public health issues in both adults and children. The etiology of obesity is multifactorial, with genetic inheritance and behavioral/environmental causes considered the most important factors leading to fat accumulation [1]. The over-consumption of highly palatable, fat-rich, energy-dense foods has been recognized as a salient environmental contributor to the increasing worldwide obesity rates.

The impact of dietary fats with different fatty acid compositions on the physiological process varies considerably. The physiological significance of fatty acids depends not only on chain length and the degree of saturation but also on the stereoisomeric configuration of double bonds [2–6]. The strong correlation between high dietary intake of industrially produced trans fatty acids (TFAs) [7–9] /saturated fatty acids (SFAs) [10] and a high risk of cardiovascular disease has been consistently demonstrated. After the US and some European countries imposed bans on TFAs, the population’s average daily intake of TFAs dramatically decreased in those countries [11,12]. However, certain subgroups of the population in certain countries and regions show high IP-TFA intake due to the increasing worldwide consumption of ultra-processed foods [13]. For instance, recent evidence indicated that although the general daily intake of TFAs was below the WHO’s recommended maximum intake as the percentage of total energy consumption in a Chinese population, the daily TFA intake of 0.42% participants did not meet the WHO recommendations [14]. In the modern food processing industry, margarine use is widespread and has been one of the main contributors to the intake of industrially produced TFAs (IP-TFAs) [15]. For IP-TFAs, it has been found that in industrially processed products, the predominant trans isomer is elaidic acid, which is derived from partially hydrogenated oils [16,17]. Elaidic acid (EA) has been widely used as a specific biomarker of margarine intake in several investigations [18,19] and sufficiently reflects the bioavailability and the subsequent harmful effects of margarine or TFAs. Consistent with the lack of changes in European SFA intake and food patterns over the past 20–25 years [12], no significant change has been observed for saturated fat intake in the US national adult representative population [11], with saturated fat accounting for one-third of the fat consumed by the US population [20]. The main dietary sources of SFAs are animal-based products, especially lard in Chinese diets, which contributes significantly to SFAs intake [21]. Moreover, in some Chinese schools in rural or poor areas, lard is still used as a cooking oil [22].

White adipose tissue (WAT), a complex endocrine organ, has intricate physiological functions in whole-body homeostasis. It can synthesize and secrete a variety of bioactive peptides, known as adipokines, that regulate numerous biological processes at both the autocrine/paracrine and endocrine levels [23]. The WAT depot is a multifaceted structure mostly consisting of adipocytes and stromovascular cells (preadipocytes, macrophages, endothelial cells, *etc*.). It has been proven that the metabolic and endocrine functions of WAT from different depots are significantly different [24,25]. In obesity, excessive adiposity disturbs the balance of the adipose organ, causing its dysregulation and subsequently producing a negative effect on health. Low-grade inflammation within WAT is proposed to link obesity with a cluster of associated metabolic diseases, and adipose tissue macrophage (ATM) infiltration is implicated in this process [26,27]. The available evidence supports WAT as a more tangible target to reverse obesity. Researchers have investigated the serious health risks of TFAs in various ways [28], and some hold similar views on the deleterious effects of lard on adipose tissue fatty acid composition and endocrine function [29–31]. Therefore, exploring and understanding the depot-specific effects of various dietary fats are essential.

High-fat-induced animal models represent the classical and most common method applied to study obesity. Rats exposed to a high-fat diet could show variable responses in weight gain and can be classified as obesity-prone (OP) or obesity-resistant (OR) [32,33]. To determine the effects of a high-margarine/lard diet on different adipose depots, high-margarine/lard diet-induced obese rats were used to explore the inflammatory responses of depot-specific WAT to a high-margarine and lard diet. Macrophage cell markers were also detected in the present study.

## Materials and methods

### Study design

**Fig 1:**
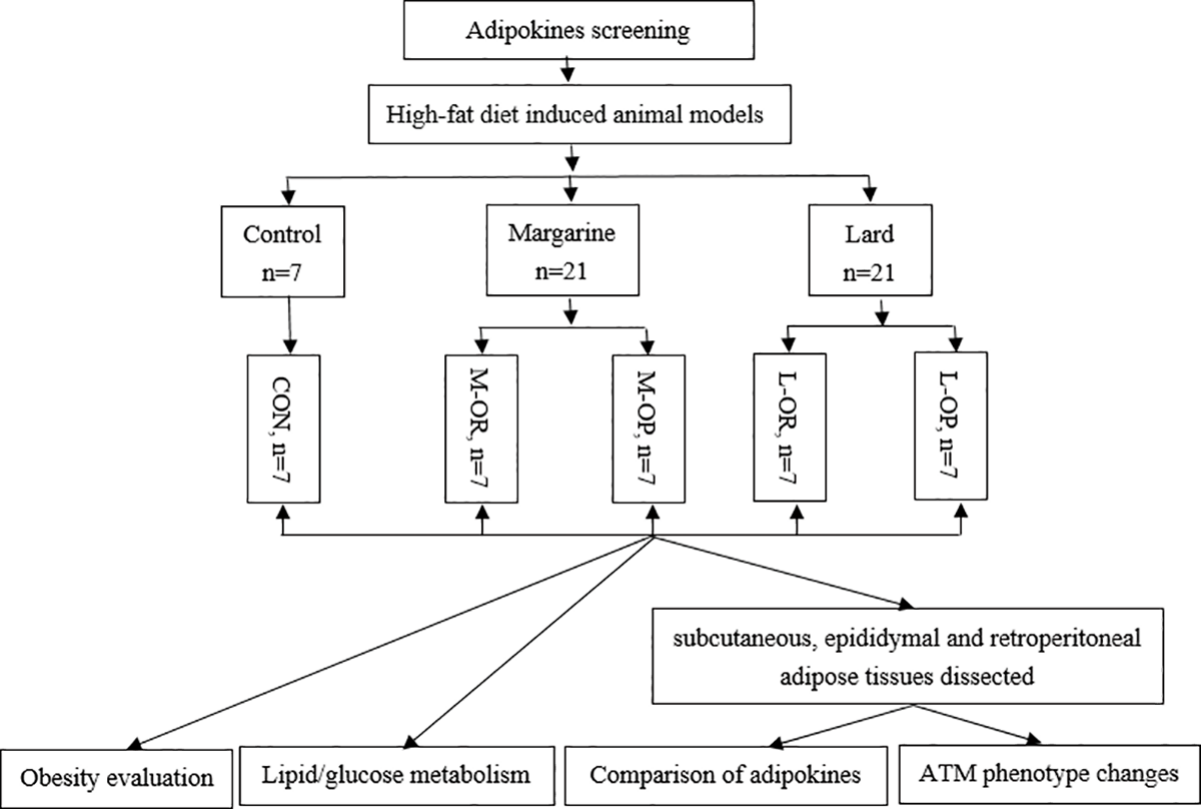
Flow of the procedures in this study.

In the present study, EA as a biomarker of margarine intake was used to screen affected adipokines on mature human adipocytes in vitro. Then, Wistar rats were fed a high-fat diet enriched with margarine/lard to generate obesity OP and OR models. The response of depot-specific WAT, adiposity and glucose and lipid metabolism parameters were compared in vivo. A flow diagram of the procedures in this study is illustrated in Fig 1.

### Culture and exposure of adipocytes

Human mesenchymal stem cells (hMSCs) were purchased from Cyagen Biosciences Inc. (HUXMD-01001) and cultured in DMEM/F-12 media with 10% heat-inactivated fetal bovine serum (FBS) and 1% penicillin/streptomycin at 37°C in 5% CO2. The cells were washed with PBS and were cultured in differentiation medium (DMEM/F-12 containing 15% FBS, 5% Rabbit serum, 20 nmol/L of human insulin, 1% penicillin/streptomycin, 1 mM of dexamethasone, 0.25 mmol/L of 3-isobutyl-1-methylxanthine (IBMX), and 5 μm of indomethacin), and then, IBMX and indomethacin were withdrawn after 3 days of exposure. After 12 to 14 days of differentiation, the adipocytes were washed with serum-free DMEM/F-12 and treated with 100 μmol/L of EA and 100 μmol/L of its isomer, oleic acid (OA). The adipocytes of the control group were treated with serum-free DMEM/F-12 (control) and 1% DMSO (vehicle), respectively. After twenty-four hours, the media and cells were collected and stored at -80°C for protein determination.

### Antibody array

After the differentiation of hMSCs, mature adipocytes were treated with 100 μmol/L of EA. Twenty-four hours later, the supernatant was discarded, and then, the adipocytes were washed with ice-cold PBS 3 times and collected and stored at -80°C. To gain insight into the variably and characteristically expressed adipokines in adipocytes, chips of RayBio Human Labelbased Antibody Array I (AAH-ADI-M) were applied to measure the expression levels of 62 adipokines simultaneously, and the presence of adipokines was identified by the Genepix 4000B Microarray Scanner (Axon Instruments, Inc., USA).

### Determination of adipokines in cell culture media

The concentrations of adipokines in cell culture media were analyzed in duplicate according to the manufacturer’s protocols with enzyme-linked immunosorbent assay (ELISA) kits from Elabscience Biotechnology Co. Ltd, Wuhan, China.

### Animal studies

Forty-nine healthy, SPF-grade weaned male Wistar rats, initially weighing 130 to 150, were obtained from the Center for Experimental Animals at China Medical University (Shenyang, China) with a National Animal Use License number of SCXK-LN2003-0009. All experiments and surgical procedures were approved by the Animal Use and Care Committee at China Medical University with a protocol number of CMU 62043006 and complied with the National Institutes of Health Guide for the Care and Use of Laboratory Animals. All efforts were made to minimize animal suffering and to reduce the number of animals used. All rats were randomly assigned into three groups: the control group (CON, n = 7), the margarine group (M, n = 21), and the lard group (L, n = 21). Rats in CON group were given normal chow containing 10% of kcal from fat, and the remaining rats were given either homemade high-margarine chow or high-lard chow. The rats were housed with a 12-h light/dark cycle and free access to food and water. After 8 weeks of feeding with their respective diets, the lower tertile rats in the high-fat groups were further divided into an obesity-resistant (OR) group, and the upper tertile rats were assigned to an obesity-prone group (OP) according to body weight gain with the help of the method previously described by Levin [32]. The rats with intermediate tertile body weight gain were excluded from this study. All sections of this report adhere to the ARRIVE Guidelines for reporting animal research [34]. A completed ARRIVE guidelines checklist is included in the supporting information (S1 Checklist).

### Diets

The margarine and lard chows contained 45% of kcal from fat and were composed of 73% standard chow diet plus 20% commercially available margarine or lard, respectively (1.5% TFAs in margarine and no TFAs in lard according to the manufacture’s indicated nutrition facts, purchased from a local supermarket), 7% casein (Aoboxing Biotech Company Ltd, Beijing, China), and trace amounts of multiple vitamins. To ensure a diet-induced effect from dietary fatty acid content, the fat-enriched diets were engineered to have identical macronutrient profiles that differed only in margarine/lard content. The detailed macronutrient profiles and energy content of the diets are shown in Table 1.

**Table 1:** Macronutrient profiles and energy content of the diets (g/kg).

Ingredients	SC	HL	HM
Protein (g/kg)	210	284	238
Carbohydrate (g/kg)	630	387	387
Fat (g/kg)	40	232	232
Protein (% energy)	23.3%	23.3%	23.3%
Carbohydrate (% energy)	66.7%	31.7%	31.7%
Fat (% energy)	10.0%	45%	45%
Energy content (kcal/kg)	3784	4877	4877

SC, standard chow; HL, high-lard diet; HM, high-margarine diet.

### Processing of tissues

At the end of the 8th week, all rats were sacrificed under anesthesia with 10% chloral hydrate after 12 h of fasting. Subcutaneous, retroperitoneal and epididymal fat tissues were immediately dissected, and the weights of retroperitoneal fat and epididymal fat were calculated using the following formula: body fat coefficient = fat weight/animal body weight×100%. Then, the tissues were immediately stored at -80°C for western blot analysis.

### Western blot analysis

Briefly, epididymal, retroperitoneal and subcutaneous fat tissues were homogenized in 200–300 μl of RIPA buffer isotonic cocktail containing protease and phosphatase inhibitors. Protein samples were boiled for 5 min in 1 × SDS sample buffer (50 mmol/L of Tris-HCl, pH 6.8, 20% glycerol, 2% SDS and 0.02% bromophenol blue) containing 2% mercaptoethanol. Samples with approximately 50 μg of proteins were subjected to SDS-PAGE using 12% polyacrylamide gels at a voltage of 100 V. The proteins on the gels were transferred onto a PVDF membrane (0.2 μm) for 20 minutes. The membrane was blocked with 5% BSA for 1 h at room temperature and incubated with Anti-IL6 Monoclonal Antibody (1:3000 dilution; abcam, Catalog Number ab9324,USA); SDF-1 Polyclonal Antibody (1:2000 dilution; ImmunoWay, Catalog NumberYT4225,USA); XCL1/Lymphotactin Polyclonal Antibody (1:2000 dilution; novusbio, Catalog NumberNBP1-45690, USA); Anti-MCP1 Polyclonal Antibody (1:2500 dilution; abcam, Catalog Number ab25124, USA); Anti-Lymphotactin Monoclonal Antibody (1:2000 dilution; abcam, Catalog Number ab122971, USA); and GAPDH (1:1000 dilution; Cell Signaling Technology, Catalog Number 2118, USA) overnight at 4°C, followed by horseradish peroxidase-conjugated secondary antibodies (1:2000 dilution; DingGuo, China) for 1 hour at room temperature. Qualitative bands were then quantified using ImageJ2x software (Version 2.1.4.7, Wayne Rasband, National Institutes of Health, USA).

### Biochemical measurements

Fasting blood samples were collected from the abdominal aorta. Serum triglycerides (TG), total cholesterol (TCHO), high-density lipoprotein cholesterol (HDL-C), low-density lipoprotein cholesterol (LDL-C), glucose (GLU), and insulin (INS) were determined using commercial kits (Beijing BHKT Clinical Reagent Co., Ltd).

### RNA extraction and RT-PCR

Total RNA in adipose tissues was extracted using the TRIzol (Invitrogen, Carlsbad, CA) isolation method and was reverse transcribed into cDNA using the PrimeScript RT reagent kit with gDNA Eraser (TaKaRa, Dalian, China) according to the manufacturer’s instructions. The gene-specific primers were designed by TaKaRa Co. The following PCR primers were used for real-time PCR: F4/80 (forward: TGCAGTTCAGAACCACAACACCTAC, reverse: CCCGCAATGATAGCGCAAG), CD206 (forward: TCGGGTGAACGGAATGATTG, reverse: AAGAGCCCTTGGGTTGAGGA), CD11c (forward: GGCTGAAATCACTTTCGACACA, reverse: GAAGATGGGCTCATAGACCACGTA) and β-actin (forward: GGAGATTACTGCCCTGGCTCCTA, reverse: GACTCATCGTACTCCTGCTTGCTG). Real-time PCR reactions were carried out using a SYBR green PCR kit (TaKaRa Biotechnology Co., Ltd., Dalian, China) on the 7500 Real-Time PCR system (Becton, Dickinson and Company, USA). Relative expression was determined by the 2^−ΔΔCT^ method. All reactions were performed in triplicate.

### Statistical analysis

The results are expressed as the mean ± SD. A one-way analysis of variance followed by the Dunnet-*t* test was used to compare the control group with the treatment groups. Spearman correlation coefficients were adopted to analyze the strengths of relationships between variables of interest.

All statistical tests were two-sided, and a *P* value less than 0.05 was considered statistically significant. All statistical analyses were conducted using the software IBM SPSS Statistics 21.0 for Windows (IBM, Asian Analytics Shanghai).

## Results

### Adipokines screening

**Fig 2:**
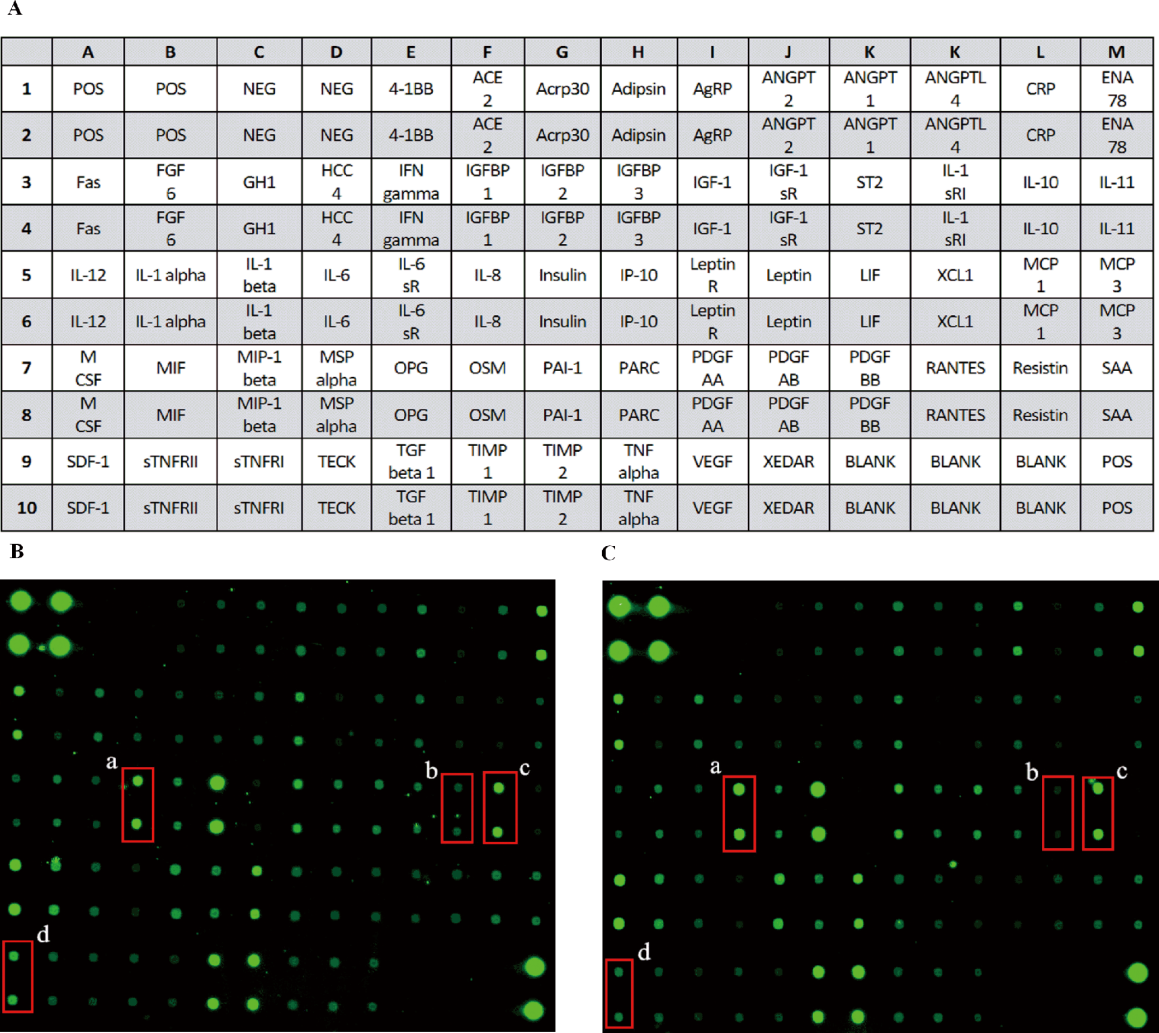
Scanning images of the antibody array analysis. **(**A) Map of antibody; **(**B) CON group; (C) EA group. a. IL-6, b. XCL1, c. MCP-1, d. SDF-1.

Scanning images of protein chips are displayed in Fig 2. The fluorescence intensity represented a protein's expression level. The fold change results of proteins indicated that the expression levels of IL-6 and MCP-1 were markedly up-regulated and that SDF-1 and XCL1 were obviously down-regulated in adipocytes exposed to EA compared with the levels of control adipocytes (Table 2). Detected fluorescence intensities and fold change data compared with the control data are provided in the supporting information (S1 Dataset).

**Table 2:** Proteins with significant expression in cultured adipocytes according to antibody array results.

	IL-6	MCP-1	SDF-1	XCL1
**CON**	488.8±27.8	797.0±44.2	290.5±28.3	167.8±65.6
**EA**	1498.8±267.5	1486.7±280.3	176.3±32.3	79.3±13.8
**Fold Change**	3.1±0.7	1.9±0.5	0.6±0.1	0.5±0.2

IL, interleukin-6; MCP-1, monocyte chemoattractant protein-1; SDF-1, stromal cell derived factor-1; XCL1, lymphotactin.

#### Verification of antibody array data via western blot analyses and ELISA assay

**Fig 3:**
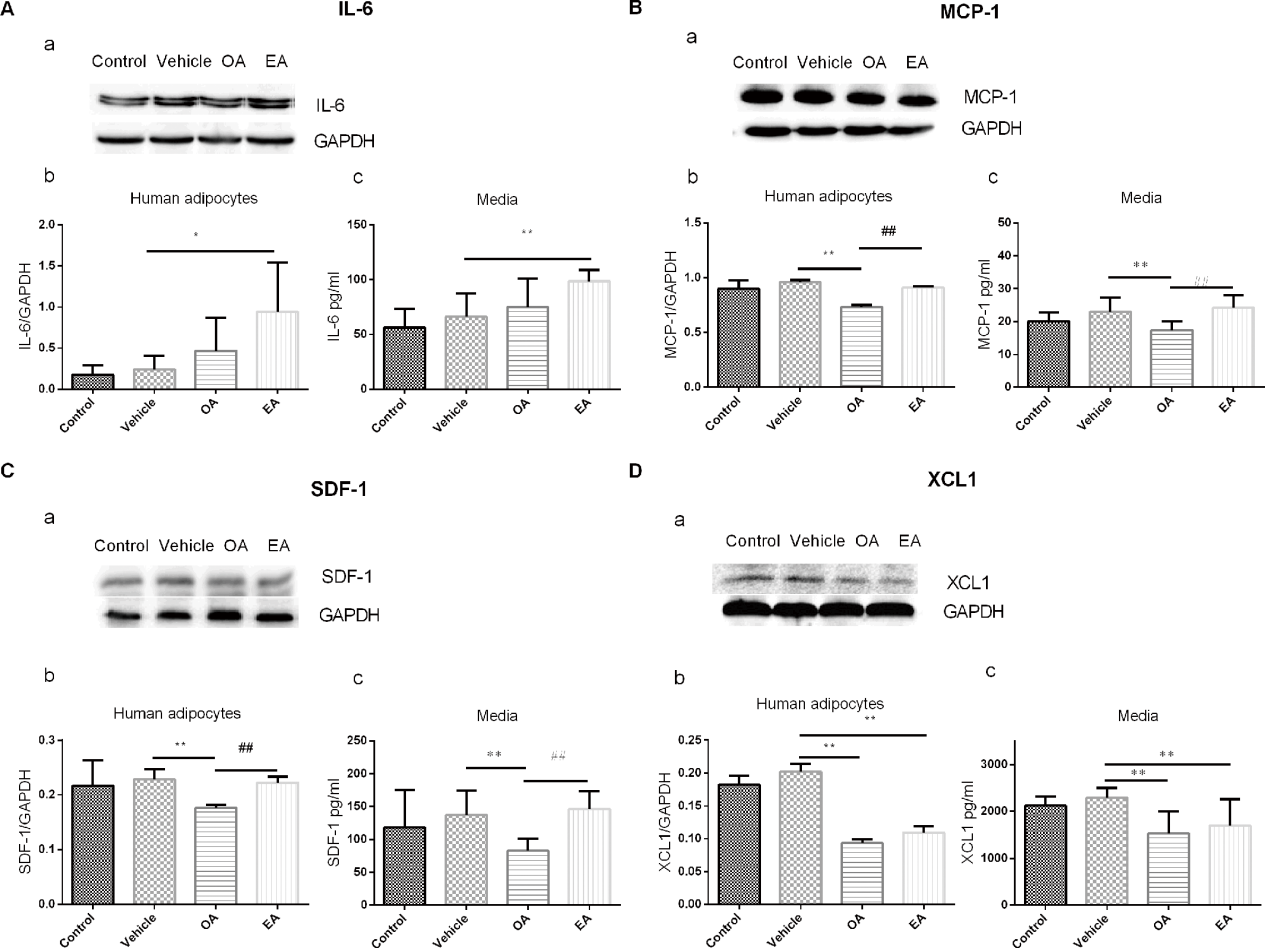
Production and secretion of specific adipokines in adipocytes. (A) IL-6; (B) MCP-1; (C) SDF-1; (D) XCL1. (a) Representative bands; (b) Protein expression in adipocytes; (c) Adipokine concentration in media. (Control: only containing DMEM/F-12; Vehicle: only containing1‰DMSO; OA: DMEM/F-12 containing 100μmol/L OA; EA: DMEM/F-12 containing100μmol/L EA. n = 6, ** *P<* 0.01, * *P<* 0.05 *vs* Vehicle group; ^##^ *P<* 0.01 *vs* OA group).

To verify the results of previous protein chips, western blot and ELISA assays were carried out to evaluate the production and secretion of IL-6, MCP-1, SDF-1 and XCL1 (Fig 3). Only EA could lead to increased protein expression of IL-6 compared with the vehicle group. The OA group exhibited lower MCP-1 expression compared with the vehicle group; however, EA led to increased MCP-1 expression compared with the OA group. SDF-1 protein expression was significantly decreased only in the OA group. Interestingly, the EA group exhibited higher SDF-1 expression compared with the OA group. However, the protein expression of XCL1 was significantly decreased in the EA and OA groups, with statistical significance.

### Different effects of high-lard/margarine diets on OP/OR rats

#### Body-weight gain and fat distribution

As shown in Table 3, the rats in the OP group that were fed either lard or margarine showed significantly greater increases in body-weight gain compared with standard chow-fed rats. After 8 weeks of feeding, rats in the M-OP/M-OR/L-OP groups showed a significant increase in epididymal and retroperitoneal fat accumulation compared with rats in the CON group. However, rats in the L-OR group only showed a significant increase in the retroperitoneal fat depot, and no significant difference was observed in the epididymal fat of L-OR rats or CON rats.

**Table 3:** Comparison of body-weight gain and body fat coefficients of rats fed with high-lard/margarine diets.

	n	Body-weight gain(g)	Epididymal fat coefficient (%)	Retroperitoneal fat coefficient (%)
**CON**	7	281.51±19.82	0.57±0.09	0.51±0.13
**L-OR**	7	276.34±20.84	0.80±0.26	0.82±0.12**
**L-OP**	7	345.77±18.56**	0.92±0.16**	1.06±0.25**
**M-OR**	7	290.19±27.98	1.12±0.15**	1.30±0.18**
**M-OP**	7	378.64±27.59**	1.19±0.38**	1.24±0.4**

CON, control group; L-OR, Lard obesity resistant group; L-OP, Lard obesity prone group; M-OR, Margarine obesity resistant group; M-OP, Margarine obesity prone group.

** *P* <0.01, compared with CON group using ANOVA.

#### Detection of serum lipid and glucose metabolism levels

As shown in Table 4, the TCHO level of the L-OR group was significantly higher compared with that of the CON group. The TCHO and HDL-C levels of the L-OP group were higher than those of the CON group, with statistical significance. Moreover, the TG and HDL-C levels in the M-OR group were significantly higher than those of the CON group. The Homeostasis model insulin resistance index (HOMA-IR) was significantly elevated in the M-OP group compared to that of the CON group, but not in the L-OR/L-OP/M-OR groups.

**Table 4:** Comparison of serum lipid and glucose metabolism measurements of rats fed with high-lard/margarine diets.

Group	n	TG (mmol/L)	TCHO (mmol/L)	LDL-C (mmol/L)	HDL-C (mmol/L)	TCHO/ HDL-C	GLU (mmol/L)	INS (mIU/L)	HOMA-IR
**CON**	7	0.28±0.03	0.95±0.13	0.63±0.10	0.49±0.07	0.24±0.08	13.57±0.59	24.01± 3.16	14.53±2.33
**L-OR**	7	0.23±0.03	1.16±0.08*	0.78±0.08	0.51±0.08	0.27±0.10	13.06±1.39	29.56± 5.02	16.98±2.08
**L-OP**	7	0.29±0.03	1.22±0.15**	0.74±0.16	0.67±0.09*	0.32±0.12	13.38±1.96	30.58± 8.01	18.47±7.31
**M-OR**	7	0.45±0.09**	1.01±0.05	0.67±0.06	0.60±0.06*	0.18±0.07	13.77±1.01	30.07±13.80	18.62±9.26
**M-OP**	7	0.30±0.07	1.03±0.21	0.72±0.15	0.52±0.11	0.31±0.12	13.39±0.86	35.00±5.47*	20.79±3.43*

CON, control group; L-OR, Lard obesity resistant group; L-OP, Lard obesity prone group; M-OR, Margarine obesity resistant group; M-OP, Margarine obesity prone group.

** *P* <0.01

* *P* <0.05, compared with CON group using ANOVA.

#### Expression of adipokines in subcutaneous, epididymal and retroperitoneal adipose tissues

**Fig 4:**
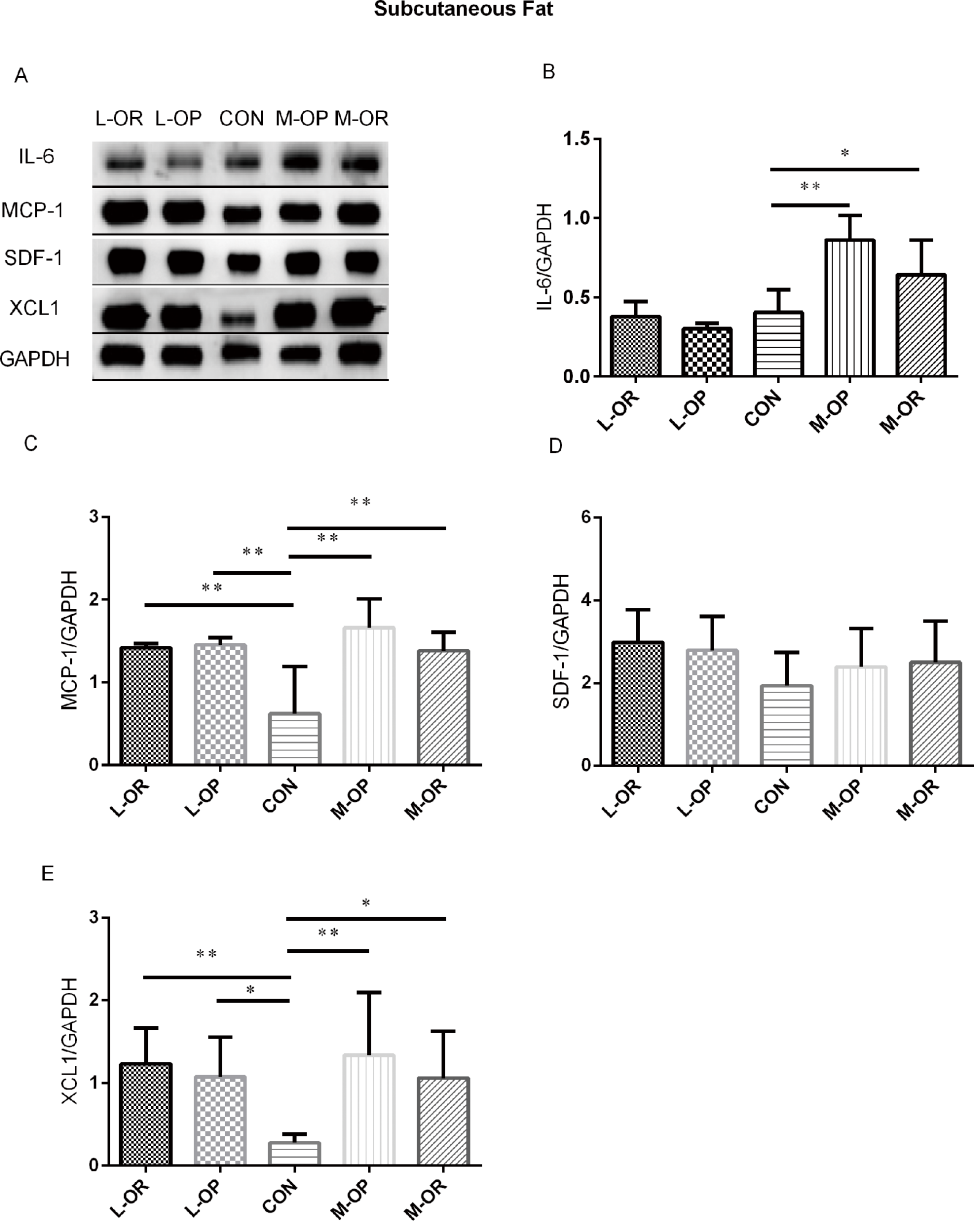
Analysis of adipokine expression in subcutaneous adipose tissue. (A) Representative IL-6, MCP-1, SDF-1, XCL1 Western blots of subcutaneous adipose tissue. (B) Quantification of the Western blot membranes for IL-6. (C) Quantification of the Western blot membranes for MCP-1. (D) Quantification of the Western blot membranes for SDF-1. (E) Quantification of the Western blot membranes for XCL1. L-OR, Lard obesity resistant group; L-OP, Lard obesity prone group; CON, control group; M-OP, Margarine obesity prone group; M-OR, Margarine obesity resistant group. All experiments had been performed in at least triplicate. n = 6. Data are presented as means ± SD. ** *P*< 0.01, * *P*< 0.05 *vs*. CON.

**Fig 5:**
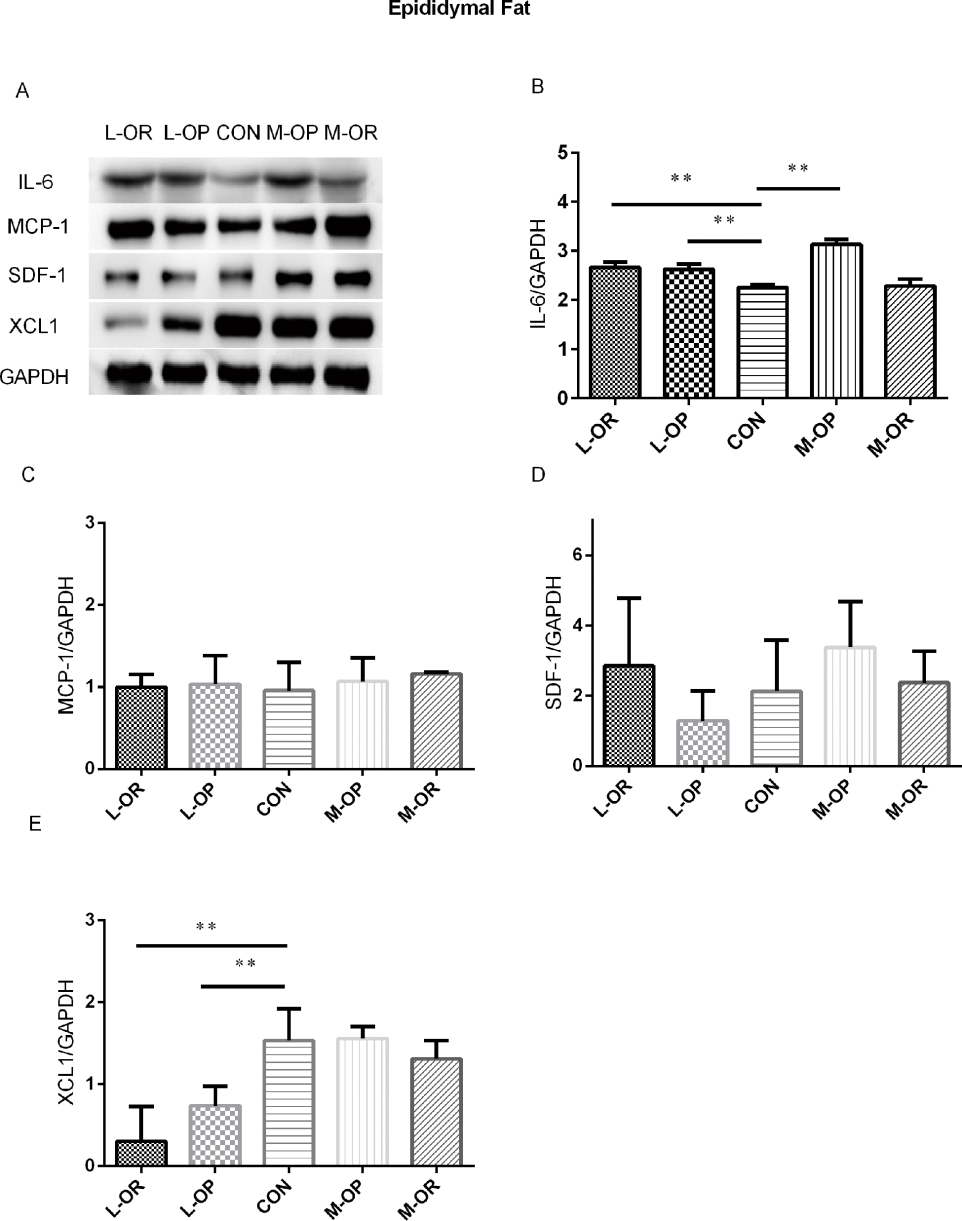
Analysis of adipokine expression in epididymal adipose tissue. (A) Representative IL-6, MCP-1, SDF-1, XCL1 Western blots of epididymal adipose tissue. (B) Quantification of the Western blot membranes for IL-6. (C) Quantification of the Western blot membranes for MCP-1. (D) Quantification of the Western blot membranes for SDF-1. (E) Quantification of the Western blot membranes for XCL1. L-OR, Lard obesity resistant group; L-OP, Lard obesity prone group; CON, control group; M-OP, Margarine obesity prone group; M-OR, Margarine obesity resistant group. All experiments had been performed in at least triplicate. n = 6. Data are presented as means ± SD. ** *P*< 0.01, * *P*< 0.05 *vs*. CON.

**Fig 6:**
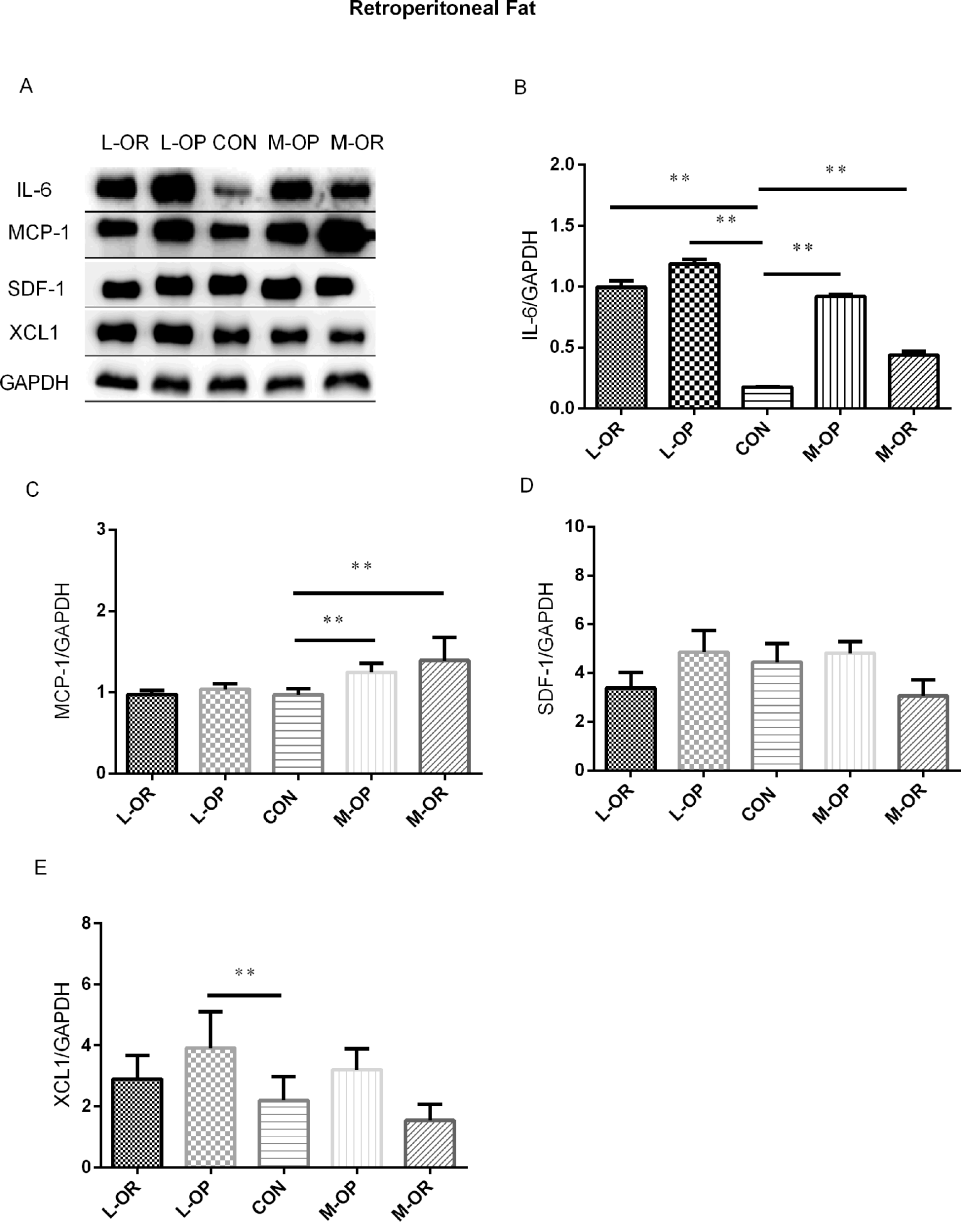
Analysis of adipokine expression in retroperitoneal adipose tissue. (A) Representative IL-6, MCP-1, SDF-1, XCL1 Western blots of epididymal adipose tissue. (B) Quantification of the Western blot membranes for IL-6. (C) Quantification of the Western blot membranes for MCP-1. (D) Quantification of the Western blot membranes for SDF-1. (E) Quantification of the Western blot membranes for XCL1. L-OR, Lard obesity resistant group; L-OP, Lard obesity prone group; CON, control group; M-OP, Margarine obesity prone group; M-OR, Margarine obesity resistant group. All experiments had been performed in at least triplicate. n = 6. Data are presented as means ± SD. ** *P*< 0.01, * *P*< 0.05 *vs*. CON.

High-lard/margarine intake was found to induce variable inflammation in subcutaneous, epididymal and retroperitoneal adipose tissues. The results are shown in Figs 4, 5 and 6, respectively.

In subcutaneous adipose tissue, a high-margarine diet induced elevated IL-6, MCP-1 and XCL1 expression levels in both the M-OP and M-OR groups. Consistently, high-lard-fed rats displayed higher protein expression levels of MCP-1 and XCL1 compared with those in the CON group. In epididymal fat depots, a high-margarine diet only elevated the level of IL-6 in the M-OP group. However, a high-lard diet induced increased IL-6 expression and decreased XCL1 expression in the L-OP/L-OR groups. In retroperitoneal adipose depots, IL-6 and MCP-1 were significantly elevated in M-OP/M-OR rats fed with a high-margarine diet. Surprisingly, although IL-6 expression was higher in L-OP/L-OR rats, the expression of XCL1 in L-OP rats was higher than that of normal chow-fed rats.

#### ATM phenotype changes in subcutaneous, epididymal and retroperitoneal adipose tissues

**Fig 7:**
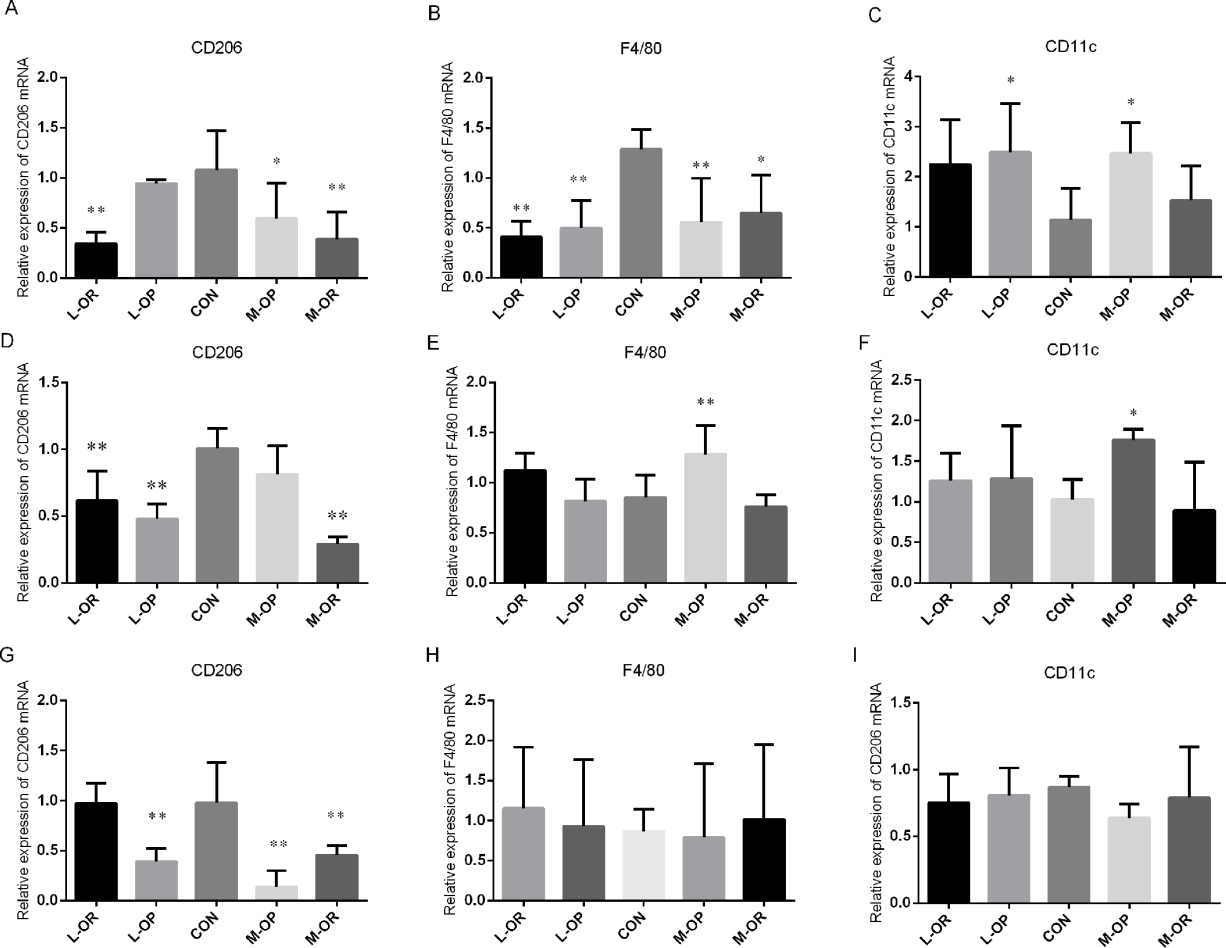
Relative mRNA expression levels of CD206, F4/80 and CD11c in different adipose depots. Relative expression of CD206 mRNA in (A) subcutaneous adipose tissue, (D) epididymal adipose tissue; and (G) retroperitoneal adipose tissue. Relative expression of F4/80 mRNA in (B) subcutaneous adipose tissue, (E) epididymal adipose tissue and (H) retroperitoneal adipose tissue. Relative expression of CD11c mRNA in (C) subcutaneous adipose tissue, (F) epididymal adipose tissue and (I) retroperitoneal adipose tissue. L-OR, Lard obesity resistant group; L-OP, Lard obesity prone group; CON, control group; M-OP, Margarine obesity prone group; M-OR, Margarine obesity resistant group. All experiments had been performed in at least triplicate. n = 6. Data are presented as means ± SD. ** *P*<0.01, * *P*<0.05 *vs*. CON.

The expression of ATM phenotypes was detected by RT-PCR as shown in Fig 7. In subcutaneous adipose tissues, CD206 mRNA levels were significantly lower in the M-OP, M-OR, and L-OR groups than those in the CON group, F4/80 mRNA levels were significantly lower in all treated groups, and CD11c mRNA levels were significantly increased in the L-OP and M-OP groups, but no significant differences were observed between the L-OR/M-OR and CON groups. In epididymal adipose depots, CD206 mRNA expression levels were markedly decreased in the L-OP/L-OR/M-OR groups compared with those in the CON group, and the F4/80 mRNA level and CD11c mRNA level were only markedly elevated in the M-OP group. In addition, the RT-PCR results showed that the expression of CD206 was down-regulated in retroperitoneal adipose depots in the L-OP/M-OP/M-OR groups, but no significant changes in F4/80 and CD11c mRNA expression levels were observed in the lard and margarine groups.

## Discussion

The occurrence of obesity is influenced by many factors, with a high-fat diet as a major contributor [35], and the types of fatty acids in food also play a significant role in the development of obesity[1,36]. Obesity is a disorder with excessive accumulation of body fat as the prominent clinical manifestation, especially in white adipose tissue [37].

Although dietary fat is one of the main sources of essential fatty acids for the body, it has been established that excessive consumption of some fatty acids results in health hazards. TFAs are known as the most harmful type of dietary fats. In addition to involvement in inflammation and cancer, the intake of high amounts of industrially produced TFAs, more specifically, the major TFAs in hydrogenated fats, including elaidic acid, is correlated with an increased risk of cardiovascular diseases and diabetes[38–40]. According to the results of an epidemiological investigation in China, more than 70% of the total dietary intake of TFAs is derived from industrially processed foods. Such a high proportion could result in serious health consequences. It has been found that the mean TFA content is highest in margarine in Chinese diets[14]. Similarly, in New Zealand, home use of margarine, which is made from partially hydrogenated vegetable oils, has been identified as major determinant of trans fatty acid exposure [41]. Considering TFA intake relative to the percentage of total energy intake, a relatively high level of dietary TFA intake has been reported in Japan and some western counties [42]. As a common source of animal fat, lard is a major contributor to the high SFA intake in different countries and regions [22,43]. In the US adolescent population, total fat and saturated fat intake exceeded the recommended maximum levels [44]. Although TFAs are not present in lard, previous evidence has revealed a significant relationship between SFA or lard and cardiovascular diseases [43,45,46]. These two types of fat, which can be found in both eastern and western food ingredients, attracted our interest in terms of the body’s reaction to them, especially in depot-specific WAT.

In the initial stage, the results of the vitro experiments provided some evidence that EA could affect the production of IL-6, MCP-1, SDF-1 and XCL1 in adipocytes. Interestingly, these agents are currently classified as inflammatory markers. More specifically, IL-6 is known as a pro-inflammatory cytokine [47], and MCP-1, XCL1, SDF-1 are regarded as chemokines [48–50]. Cytokines and chemokines are usually involved in obesity-associated insulin resistance, nonalcoholic fatty liver disease, and the development of allergies and autoimmune diseases [51,52]. Fluctuations of these peptides’ production can significantly increase the risks of diseases.

The subsequent western blot results verified adipocytes’ dissimilar responses to EA compared with its isomer OA. Compared with the control/vehicle groups, EA markedly increased IL-6, MCP-1 and SDF-1 production and lowered XCL1 production compared with OA. Adipose tissue as an endocrine organ plays a role in metabolic regulation [53], and fatty acid exposure from the environment could disturb the balance of adipocytes. The ELISA results from the cell culture media indicated that aberrant adipokine release due to adipose tissue dysfunction may contribute to the pathogenesis of obesity-linked complications [54]. Our results confirmed that exposure to cis or trans fatty acids could induce inflammatory effects on adipocytes, which is consistent with the notion that obesity reflects a state of low-grade inflammation [55]. Furthermore, trans fatty acids, such as EA, could induce even stronger inflammatory effects than their cis isomers.

It is necessary to observe the biological effects of fatty acids on white adipose tissues in the whole body. High-fat-induced animal models represent an important tool for studying the physiological and molecular events in the development of obesity as they share similar global gene expression patterns with obese humans [31,56–58]. High-fat diet-induced obese rats were used to explore the specific responses of different white adipose depots to margarine and lard intake in our study.

Body-weight gain is the basis of obesity diagnosis. As previously reported, similar energy densities, but different types of fatty acids, could lead to the same obesity outcomes, but with different physical and molecular effects on experimental rats [1]. Researchers have reported that Wistar rats show the OP or OR phenotype when exposed to a high-fat diet [32,33]. In the present study, OP rats fed with high-margarine/lard diets gained significantly more body weight than CON group rats after 8 weeks. Although the rats in the OR groups showed similar weight gains to that of the CON group, the fat coefficients of epididymal and retroperitoneal sites indicated that OR rats were more obese than their CON counterparts. Consistent with our observation, several previous studies showed no effect of fat type on the development of obesity relative to high-fat diets based on mammal or plant fats, although some reports have described no variations in body weight and less hypertrophy of visceral fat depots with fish oil-based diets [59,60]. Taken together, these results suggest that high-margarine/lard diets could prompt body fat accumulation and eventually lead to obesity. Moreover, obesity-resistant individuals with moderate figures and undiscerning body-weights should be especially vigilant in terms of dietary fat intake as imperceptible health damage may have already occurred.

In obese rats, we observed detrimental effects of high-margarine/lard diets on metabolism. Our data illustrated that a high-lard diet could induce dyslipidemia rather than abnormal glucose metabolism. However, high-margarine diet rats showed notable differences compared with CON rats, exhibiting dyslipidemia, elevated TG levels, and insulin resistance as represented by the HOMA-IR. These results suggest that people who ingest excessive amounts of lard should be aware of potential dyslipidemia and that people with a high margarine intake should monitor their glucose and lipid metabolism markers frequently. Most dietary recommendations that focus on reducing TFA and SFA intake were confirmed to be accurate when applied to the prevention of chronic diseases such as cardiovascular disease and diabetes.

As a powerful endocrine organ, adipose tissue can secrete a variety of hormones, chemokines and adipokines, which are key mediators linking adipocytes and adipose tissue macrophages and regulating adipose tissue inflammation [61]. There are anatomical, cellular, molecular, physiological and metabolic differences in different WAT depots [24,25], and these depot-specific differences could provide useful information regarding the mechanisms resulting in diverse depot-specific functions. Considering our adipocyte results, we focused on the production of inflammatory proteins in specific fat depots of rats that were fed with enriched margarine/lard diets. As expected, different fat depots showed unsynchronized inflammatory responses to margarine/lard diets.

In subcutaneous adipose depots, IL-6 production was elevated in the M-OP and M-OR groups. The body fat analysis proved that both M-OP and M-OR rats were obese and confirmed the previous finding of increased IL-6 mRNA expression in subcutaneous adipose tissues in obese individuals compared to lean or overweight individuals [62]. These results were not observed in the lard-treated group, suggesting that margarine could induce more severe inflammatory responses than lard. IL-6, due to its pro-inflammatory activity, is known to increase the risks of various kinds of cancers in obese patients, such as breast, liver, prostate, colon, and esophagus cancers [63]. Some epidemiological investigations found that margarine intake was positively associated with prostate cancer [64]. Recent retrospective studies reported higher margarine intake as an inflammatory dietary pattern that may increase the incidence of premenopausal breast cancer [65]. We speculate that increased IL-6 production in subcutaneous adipose tissue may contribute to the increased risks of cancers described above. MCP-1 was increased in both margarine- and lard-treated rats in the OP and OR groups, supporting the results from Sindhu et al. that showed that obesity was positively correlated with MCP-1 gene expression in subcutaneous adipose tissue [62]. Moreover, researchers have found that MCP-1 mRNA in patients with type 2 diabetes is up-regulated compared with that of non-diabetic subjects in subcutaneous adipose tissues [66]. Considering the wide distribution of subcutaneous fat in the body, increased high-fat-induced MCP-1 production could be one of the causes of obesity-associated insulin resistance. XCL1, a C class chemokine also known as lymphotactin, is produced by T, NK, and NKT cells during infectious and inflammatory responses [67–69]. Its specific chemotactic properties on lymphocytes and neutrophils have been extensively studied and explained [67,70,71]. Existing reports on XCL1 have shown its potent roles in the occurrence and development of carcinomas [72], mycobacterium tuberculosis infection [73], HIV infection [74], allergic inflammation [75] and nerve injury [76]. We found that XCL1 production was elevated in the margarine/lard diet groups, which is consistent with Skopková M‘s results that obese subjects had higher lymphotactin levels in biopsies of subcutaneous adipose tissues [77]. We speculate that exposure time to fatty acids may be a significant factor in the development of obesity and that elevated XCL1 production may be attributed to systemic coordination in an obese state.

In our study, both a high-margarine diet and a high-lard diet could cause inflammation in epididymal adipose tissue, but the effects did not exactly follow the same pattern. We found that IL-6 production was significantly increased in the L-OP, L-OR, M-OP groups, and similar results have also been described in other high-lard-induced obesity studies in mice [78], suggesting that IL-6 could serve as a marker of high-fat diet intake in epididymal adipose depots. In contrast to elevated XCL1 expression in subcutaneous depots, we observed that XCL1 production was decreased in epididymal depots in high-lard diet-fed L-OR and L-OP rats compared with CON rats. Recent experimental results showed that XCL1 was significantly down-regulated in the endometrium of obese infertile patients [79], suggesting that XCL1 expression may exhibit depot-specific characteristics. Further prospective studies in broader populations are required to understand the detrimental effects of XCL1 on reproduction. Since epididymal fat is necessary for spermatogenesis [80], we support the hypothesis that a high level of IL-6 in epididymal adipose tissue could be a fundamental cause of reproductive function damage induced by a high-fat diet in obese males. However, more experiments should be carried out to determine the relevant relationship and mechanism. A recent investigation demonstrated that a diet containing 60.3% of energy from lard could elevate epididymal MCP-1 gene expression in mice [81], which was not observed in our study. This discrepancy could be explained by the distinctive contribution of dietary fat to energy as a high percentage of dietary fat could interfere with MCP-1 production in epididymal adipose depots.

The role of retroperitoneal adipose tissue in kidney disease has received increasing attention. Existing evidence suggests that perirenal fat thickness is an independent predictor of chronic kidney disease in type-2 diabetic patients [82]. We found that high dietary fat intake could lead to faster growth of retroperitoneal fat pads compared to normal dietary fat intake. Moreover, Luo et al recently reported that a high-fat diet induced renal inflammation and increases in the expression of inflammatory cytokines, including as MCP-1, IL-1β, IL-6, and TNF-α [83]. Our data showed that IL-6 was elevated in both the margarine and lard diet groups, suggesting that IL-6 is also sensitive to a high-fat diet in retroperitoneal adipose depots. A high-lard diet could elevate XCL1 production more than a high-margarine diet in L-OP rats, but only slight differences were observed among the L-OR, M-OP, M-OR and CON groups. It has been found that XCL1 is involved in the positive regulation of the extracellular signal-regulated kinases pathway, which has been shown to be activated by adipogenic stimuli such as insulin, resulting in adipocyte hypertrophy and the recruitment of new adipocytes through the differentiation and development of insulin resistance in obesity [84]. A high-margarine diet could induce MCP-1 protein over-expression compared to a high-lard diet. Obesity-induced glomerulopathy is known to be associated with the upregulation of key inflammatory mediators [85], and high dietary fat intake is a major risk factor for the development of metabolic and cardiovascular-renal dysfunction [86]. MCP-1 has been proven to participate in the pathogenesis of chronic renal injury in obesity [87]. With the aim of identifying biomarkers of renal fibrosis to help predict disease progression, a recent systematic review concluded that MCP-1 may identify patients at risk of developing renal fibrosis and having a worse prognosis [88]. Our findings indicated that inflammation of retroperitoneal adipose tissue may contribute to the renal damage induced by high-lard/margarine diets.

SDF-1 is a chemokine and a potent chemoattractant for T cells and monocytes that is highly expressed in endothelial cells. It was recently identified as an adipocyte-derived chemokine (C-X-C motif) ligand 12, also known as CXCL12 [89]. Diverse manifestations of SDF-1 changes in obesity have been observed in both animal and human studies. Investigators have revealed a positive correlation between SDF-1 levels and BMI in humans [90]. However, a linear regression analysis showed a significant negative correlation between plasma SDF-1 concentration and waist circumference in adolescents [91]. High-calorie diet-induced obese mice displayed decreased concentrations of SDF-1 [92]. In contrast, obesity induced with a high-fat diet for 20 weeks in rats resulted in a robust increase in CXCL12 expression in WAT [89]. We also observed that exposure to both EA and OA could reduce SDF-1 production in adipocytes in vitro. Although a significant trend was not present, we speculated that a short duration of high fat intake could explain this observation.

Diet-induced obesity alters adipose tissue beyond increases in depot size. Persistent adipose expansion alters cell composition, causes depot dysregulation and subsequently modifies adipocyte biology and function. Recently, researchers noted the role of adipose tissue macrophage (ATM) infiltration in obesity. Inflammation within adipose tissue is recognized as a link between obesity and a cluster of associated metabolic diseases [93]. In adipose tissue, macrophages, as the most abundant resident leukocyte, represent a central lineage through which overnutrition-associated inflammatory signals take effect [94]. Macrophages are generally classified as classically activated (M1) or alternatively activated (M2). ATM1s produce large amounts of pro-inflammatory mediators such as TNF-α, IL-1β, and IL-6, which can cause insulin resistance in adipose tissue [95]. ATM2s promote local insulin sensitivity through the production of anti-inflammatory cytokines [96]. The phenotypic switch in macrophage polarization elicited by diet-induced obesity is widely apparent [95]. While most studies have found that macrophages predominantly exhibit a more M2-like phenotype, which expresses CD206 [97], under lean conditions, fewer cells express the M1-like phenotype, which expresses F4/80. In an obese state, cells in adipose tissues express more F4/80 and CD11c [98]. Most studies have demonstrated that ATMs are responsible for increased pro-inflammatory cytokines and may contribute to obesity-associated inflammation, insulin resistance, and metabolic dysfunction [99,100]. However, Kang et al.’s recent results suggested that changes in the production of inflammatory biomolecules precede increased immune cell infiltration and the induction of a macrophage phenotypic switch in omental adipose tissues of obese patients with early metabolic dysfunction [101]. Considering that the role of ATM infiltration in obesity is still controversial, macrophage cell markers were detected to determine inflammation in different adipose depots in the present study. From our data, it is difficult to conclude which effect, infiltration or inflammation, occurred first. We found that CD206 expression generally decreased in three WATs of high-fat diet-fed rats, indicating that high-margarine/lard diets caused impaired anti-inflammatory abilities, with different depot-specific inflammation in WATs. Although the expression of CD11c increased, typical ATM1s were observed in subcutaneous and epididymal depots under high-margarine/lard diets, but the expression of another ATM1 biomarker, F4/80, did not follow a similar pattern but instead showed a similar pattern to that of CD206 in subcutaneous depots. Correlation analyses were conducted, but no significant relationship was found between F4/80 expression and adiposity or adipokine expression (as shown in supplemental data file S1 Table). Interestingly, researchers also found that high-fat diets induced homodromous mRNA levels of F4/80 and CD206 in the epididymal WAT of mice [102]. According to relevant statistics, CD11c+-recruited macrophages account for most of the increase in ATMs in obesity [103,104], and more than 90% of recruited monocytes become CD11c+ ATMs. We speculated that pro-inflammatory effects could be neutralized and that the impaired anti-inflammatory capacity was predominantly reflected by CD206 levels.

There are several limitations in the present study that need to be noted. The major limitation is that as the isomer of EA, oleic acid, was not included in the animal studies because biological correlations have been confirmed between olive oil intake and plasma oleic acid concentration [18]. Considering that lard and margarine are more common dietary fat sources than olive oil in most countries and regions, we carried out our study as described above. The second limitation in the present study is the intervention duration. An experiment with a longer intervention period is required to deeply understand the systemic effect of margarine and lard intake on health since dietary patterns are difficult to modify once established. Third, mRNA levels were assessed without using FACS analysis and more members of the ATM marker profile need to be detected.

## Conclusions

In summary, we observed that a high-fat diet could induce obesity outcomes with different phenotypes, including OP or OR, with relatively similar inflammation in individual white adipose depots in these two phenotypes. Our findings revealed that high margarine/lard dietary intake could induce depot-specific inflammation with decreased ATM2 expression in white adipose tissues.

## Supporting information

S1 Checklist ARRIVE guidelines checklist. (PDF)

S1 Dataset Detected fluorescence intensity and fold change data. (XLSX)

S1 Table Correlation analysis results. (XLSX)
